# Air Pollution: Role of Extracellular Vesicles-Derived Non-Coding RNAs in Environmental Stress Response

**DOI:** 10.3390/cells12111498

**Published:** 2023-05-29

**Authors:** Giuseppa D’Amico, Radha Santonocito, Alessandra Maria Vitale, Federica Scalia, Antonella Marino Gammazza, Claudia Campanella, Fabio Bucchieri, Francesco Cappello, Celeste Caruso Bavisotto

**Affiliations:** 1 Section of Human Anatomy and Histology, Department of Biomedicine, Neuroscience and Advanced Diagnostics (BIND), University of Palermo, 90133 Palermo, Italy; giuseppa.damico01@unipa.it (G.D.); radha.santonocito@unipa.it (R.S.); alessandramaria.vitale@unipa.it (A.M.V.); federica.scalia02@unipa.it (F.S.); antonella.marinogammazza@unipa.it (A.M.G.); claudia.campanella@unipa.it (C.C.); fabio.bucchieri@unipa.it (F.B.); francesco.cappello@unipa.it (F.C.); 2 Euro-Mediterranean Institute of Science and Technology (IEMEST), 90139 Palermo, Italy

**Keywords:** air pollution, stress, heat shock proteins, non-coding RNAs, extracellular vesicles, liquid biopsy, biomarkers, personalized medicine

## Abstract

Air pollution has increased over the years, causing a negative impact on society due to the many health-related problems it can contribute to. Although the type and extent of air pollutants are known, the molecular mechanisms underlying the induction of negative effects on the human body remain unclear. Emerging evidence suggests the crucial involvement of different molecular mediators in inflammation and oxidative stress in air pollution-induced disorders. Among these, non-coding RNAs (ncRNAs) carried by extracellular vesicles (EVs) may play an essential role in gene regulation of the cell stress response in pollutant-induced multiorgan disorders. This review highlights EV-transported ncRNAs’ roles in physiological and pathological conditions, such as the development of cancer and respiratory, neurodegenerative, and cardiovascular diseases following exposure to various environmental stressors.

## 1. Discovery and Classification of Non-Coding RNAs

Between the late 1950s and early 1960s, Crick, Jacob, and Monod devised the “central dogma” of molecular biology, stating that genetic information flows only in one direction, from DNA to RNA and RNA to protein [1,2,3]. However, over the past decades, many exceptions to this dogma have been reported demonstrating that, even if almost all the human genome is actively transcribed, only ~1.5% of it is made up of protein-coding genes, which are first transcribed into messenger RNAs (mRNAs) [4,5,6,7]. The rest of the transcribed elements were collectively called non-coding RNAs (ncRNAs) [8], which were initially considered “junk” RNA with unknown functionality. However, this concept was rapidly withdrawn since increasing evidence demonstrated the involvement of ncRNAs in multiple critical biological events, such as development, differentiation, cellular homeostasis, and the onset of disease [9,10,11]. ncRNAs are mainly distinguished into two main classes: housekeeping and regulatory ncRNAs (Table 1). The first ones, constitutively expressed in all cell types and necessary for cell viability, are ribosomal RNAs (rRNAs), transfer RNAs (tRNAs), small nuclear RNAs (snRNAs), small nucleolar RNAs (snoRNAs), and telomerase RNAs (TER) [12]. In addition to housekeeping ncRNAs, an increasing number of regulatory ncRNAs have been identified and characterized thanks to the rapid development of in-depth and high-throughput transcriptome sequencing techniques. Regulatory ncRNAs function as regulators of gene expression at different levels: epigenetic, transcriptional, and post-transcriptional; and are classified according to their size into small ncRNAs (sncRNAs) that have less than 200 nucleotides (nts), such as microRNAs (miRNAs), small interfering RNAs (siRNAs), piwi-interacting RNAs (piRNAs), and long ncRNAs (lncRNAs) that have more than 200 nts [11,12] (Table 1). The first evidence that RNA may function as a regulator of gene expression came from experiments with prokaryotes (bacteria) over forty years ago, in the 1980s, when the Escherichia coli regulatory micF ncRNA was first identified, and its abilities to hybridize with a complementary target mRNA (ompF mRNA) and to inhibit its translation were described. It was the first RNA-regulating gene expression through sense-antisense base pairing to be reported [13,14,15]. However, ncRNAs represent only a tiny fraction of prokaryotes’ genomes, most containing protein-coding sequences [8]. Instead, the genome of higher multicellular eukaryotic organisms has fewer protein-coding sequences that diminish with increasing complexity. On the contrary, there is a large amount of non-coding intergenic and intronic sequences, which are also transcribed [16,17].

## 2. Biogenesis and Role of Regulatory ncRNAs under Physiological Conditions

Regulatory ncRNAs control various physiological pathways, such as development, differentiation, and cellular homeostasis.

siRNAs form a class of double-stranded (ds) RNA molecules (~20–25 nts) able to silence target genes post-transcriptionally. Based on their origin, they can be divided into two subcategories: exogenous siRNA (exo-siRNA), originating from exogenous ds-nucleic acid resulting from an artificial insertion or virus infections, and endogenous siRNAs (endo-siRNA), originating from the endogenous genomic locus, and transcribed from transposon elements [18,19]. In both cases, the precursor dsRNA is enzymatically processed to produce a smaller ds-siRNA, which is loaded in a RISC complex and then unwound, leading to the formation of a functional RISC, mediating the target mRNA cleavage and degradation, ultimately resulting in gene silencing [20].

miRNAs, the most studied and abundant class of small ncRNAs, are endogenous short non-coding single-stranded (ss)-RNAs (~22 nts), which negatively regulate the expression of hundreds of target genes post-transcriptionally by affecting either the stability or the translation of their mRNAs [21]. Similarly to endo-siRNAs, they originate as longer primary transcripts (pri-miRNAs), which undergo two highly regulated cleavage events, resulting in the formation of a miRNA-induced silencing complex (miRISC), targeting a specific mRNA and causing mRNA cleavage or translation inhibition [22,23,24,25].

To date, thousands of miRNAs from different organisms have been identified. In humans, over 2000 miRNAs have been annotated and validated, which regulate many protein-coding genes, and, thus, many biological events, both in normal cellular homeostasis and disease [26,27,28,29,30]. The critical role of miRNAs in cell differentiation and organisms’ development appeared clear ever since the discovery of the first miRNA, controlling the timing of larval development in C. elegans [31,32,33]. One of the earliest developmental events is the switch from pluripotent to lineage-specified cells, achieved through the downregulation of pluripotency markers, decreased self-renewal potential, and the subsequent activation of a lineage-specific gene expression pattern. All these changes are orchestrated and regulated by various miRNAs. DGCR8 depletion in embryonic stem cells hinders the downregulation of pluripotency markers and allows the expression of lineage-specific genes [34]. In this regard, in humans, the miR-302 family members are exclusively expressed during gastrulation and play a key role in promoting the mesendoderm formation and suppressing the neuroectoderm lineage by acting on LEFTY1 and LEFTY2 regulatory factors of the Nodal signaling pathway [35]. Many identified miRNAs have been found expressed at specific differentiation and developmental stages of certain cells and tissues, suggesting their involvement in these pathways. For instance, several miRNAs are exclusively or highly expressed in the brain tissue. The simultaneous overexpression of four of them (miR-124a, miR-9, miR-125b, and miR-22) in neuronal progenitor cells reduced the number of astroglia-like cells and increased that of neuronal cells. It was suggested that this event might result from the miRNAs-driven downregulation of non-neuronal transcripts [36]. Particularly, miR-9 has been found to indirectly modulate the phosphorylation status of STAT3, an important intracellular signaling molecule mediating the inhibition of neuronal terminal differentiation [36,37]. Among the first miRNAs to be identified as major regulators of differentiation and lineage determination were those regulating the formation of skeletal muscles [38,39,40]. Among these are miR-1 and miR-133, which were defined as MyomiRs since they are both uniquely expressed in skeletal and cardiac muscle cells, under the control of myogenic transcriptional factors, and cooperate to finely regulate their proliferation and differentiation from myoblast precursors [41,42,43].

piRNAs form a novel class of small silencing RNAs (26–31 nts) with distinct features from known miRNAs and siRNAs. They were first reported in 2001 in Drosophila melanogaster testes and subsequently in mammals’ germline and be involved in germline stem cell maintenance and self-renewal [44,45,46,47]. However, emerging evidence has demonstrated that they are also expressed in a tissue-specific manner in multiple human somatic tissues other than the germline [48,49]. A large fraction of piRNA precursors is produced from discrete genetic loci named piRNA clusters, which can be transcribed in one direction or convergently from two ends (unidirectional or bidirectional clusters). Instead, a smaller fraction of piRNA precursors are generated from the 3′ UTR of protein-coding genes or individual transposons [50,51,52]. piRNA precursors are then processed by enzymatic machinery different from that involved in the processing of miRNA and siRNA through two different mechanisms: the primary synthesis mechanism and the ‘ping-pong’ amplification mechanism [50,53]. Once formed, mature piRNAs can bind the piwi proteins of the Argonaute family to form a piRNA/piwi complex, which is involved in germ stem cell maintenance and self-renewal by mediating chromatin rearrangement, silencing of transposon transcription, and suppression of translation [53,54,55,56,57].

lncRNAs are a class of regulatory ncRNAs that has attracted particular interest recently, first discovered in the early 1990s [58,59,60]. They can be classified according to various criteria, like their genomic origins (sense, antisense, bidirectional, intronic, and intergenic lncRNAs), their function, their subcellular localization, and so on [61]. lncRNAs are generated through pathways like that of protein-coding genes and undergo similar post-transcriptional processing (possess a 5′ capping and a 3′ poly-A tail and may be alternatively spliced) [62]. In humans, recent annotations have reported between 30,000 and 60,000 lncRNAs [63,64,65], involved in a broad spectrum of biological processes, including cell proliferation, apoptosis, and cell differentiation [65,66,67,68,69,70], through the regulation of gene expression at both a transcriptional and post-transcriptional level. In addition, many lncRNAs can induce epigenetic modifications by recruiting chromatin-remodeling complexes, such as the polycomb repressive complex 2 or other chromatin-modifying proteins [71,72,73]. Some lncRNAs, such as Evf2, act as co-factors, regulating the activity of transcriptional factors and, thus, the expression of target genes [74]. At a post-transcriptional level, lncRNAs have been demonstrated to regulate target mRNA processing, such as capping, splicing, editing, transport, translation, degradation, and stability [75].

## 3. Cellular Response to Stress and Air Pollutants

Cells are continuously exposed to multiple intrinsic or extrinsic stimuli that evoke a specific biological response known as stress. Stress is an environmental stimulus that differs from optimal conditions and influences cell growth and development. Cells respond to stress with adaptive responses to maintain homeostasis [76]. Several molecular mediators participate in the stress response, such as molecular chaperones, whose activity can be regulated by ncRNAs. Chaperones are ubiquitous proteins highly conserved throughout evolution, from bacteria to humans [77]. Many of them are also known as Heat Shock Proteins (Hsps) and can be classified according to their molecular weight: small heat shock proteins (sHsps) family from 10 to 30 kDa; Hsp40 family (40 kDa); Hsp60 (or chaperonins) with a molecular weight close to 60 kDa; Hsp70 family (70 kDa); Hsp90 family (83–90 kDa); and Hsp100/110 family with a molecular weight equal to or higher than 100 kDa [78]. They are essential for controlling protein homeostasis and maintaining the correct and functional conformation of proteins and nascent polypeptides, preventing their aggregation and misfolding [79]. Other functions of Hsps are regulating gene expression and DNA replication, cell differentiation, signal transduction, inflammatory response, carcinogenesis, senescence, and cell apoptosis [80,81,82]. The main factor involved in the heat shock response is heat shock factor 1 (HSF1), which regulates the expression of Hsps [83]. Under stress conditions, besides changing gene expression levels, the HSF1 induces several pre-translational, co-translational, and post-translational processes, likely through the involvement of some ncRNAs [84,85]. Therefore, ncRNAs participate in stress response [86,87]. Moreover, exposure to various stressors may alter their expression, with critical physio-pathological implications [88,89].

Air pollution is a main external stimulus that evokes a stress response. Oxidative stress is at the root of the toxic effect of air pollutants; it triggers several signaling pathways, such as the inflammatory response and the production of proinflammatory cytokines [90]. In addition, oxidative stress leads to the generation of reactive oxygen species (ROS), alteration of mitochondrial function and oxidative DNA damage [91,92]. The damage caused by air pollution affects almost every organ in the body, causing or contributing to many diseases [93].

Environmental pollution has several sources, both anthropogenic and natural, such as the use of fossil fuels in electricity generation, transport, industry, and housing; industrial processes and solvent use; agriculture; waste treatment; volcanic eruptions, airborne dust, and emissions of volatile organic compounds (VOCs). Air pollution can be distinguished into outdoor and indoor pollution. The latter is especially important because people in developed countries now spend most of their time (>80%) in enclosed spaces (e.g., home, school, office, public buildings or means of transport). Common indoor pollutants include environmental tobacco smoke, particulate matter (PM), nitrogen dioxide, carbon monoxide, VOCs, and biological allergens. Indoor air pollution can increase the risk of irritative phenomena, allergic sensitization, and acute and chronic respiratory disorders [94].

Air pollutants modify the normal chemical composition of the air and can be divided into primary and secondary pollutants. The former is emitted into the air as they are, such as carbon monoxide (CO), nitrogen oxide (NOx), sulfur dioxide (SO2), VOCs and particulate matter (PM). PM, a mixture of solid particles and liquid droplets, can, in turn, be divided into fractions based on the particle size, known as the equivalent aerodynamic diameter (AED), e.g., PM 10, PM 2.5 and Ultra-Fine Particulate Matter (UFPM). Particles larger than 10 μm are unlikely to enter the lower airways as most of them will be filtered by the nose and upper airways, whereas PM 10, PM 2.5 and UFPM usually reach the lower airways [95]. SO2 is produced by volcanoes and various industrial processes. Coal and oil usually contain sulfur compounds, so their combustion generates sulfur dioxide and, thus, acid precipitation. This is one of the reasons for worrying about the environmental impact of using these fuels as energy sources. CO is a gas formed from the incomplete combustion of fuels such as propane, natural gas, petrol, oil, coal or wood. Sources of carbon monoxide are devices such as furnaces, gas water heaters, wood stoves and other fuel-burning appliances that do not work efficiently, clogged chimneys, car exhaust fumes and tobacco smoke, and various industrial processes. In addition, CO is released from erupting volcanoes, forest fires, swamp gas and seaweed [96,97]. Natural sources of NOx include the activity of nitrogen-consuming microorganisms in the soil and the increased worldwide use of fertilizers in recent decades. The anthropogenic sources of NOx are motor vehicles (49%), electric utilities (27%), other industrial, commercial and residential sources (19%) and all other activities (5%) that burn fuel [98]. Natural VOC emissions arise from vegetation and the degradation of organic material; anthropogenic emissions, on the other hand, are mainly from the incomplete combustion of hydrocarbons and the evaporation of solvents and fuels [99].

Secondary pollutants are subsequently formed in the atmosphere because of chemical reactions involving the primary pollutants and sometimes the natural components of the atmosphere. Among the processes involved in the formation of secondary pollutants, there is photochemical smog, which consists of a series of reactions between nitrogen oxides and hydrocarbons in the presence of UV light. This series of reactions results in the oxidation of nitrogen monoxide (NO) to nitrogen dioxide (NO2), the production of ozone (O3) and the oxidation of hydrocarbons, with the formation of peroxyacetyl nitrate (PAN), formaldehyde, nitric acid, nitrates, and nitro-derivatives in the particle phase [100]. Other vital mechanisms leading to secondary pollutants are oxidation reactions and secondary organic aerosols (SOA) formation. The former occurs when primary pollutants react with oxygen in the atmosphere. For example, sulfur dioxide can be oxidized to form sulfuric acid, which can contribute to the formation of acid rain. Secondary organic aerosol formation occurs through the condensation of gases in the atmosphere [101,102]. When primary pollutants, such as VOCs, react with other atmospheric chemicals, they can form small particles known as aerosols. These aerosols can then condense to form larger particles, contributing to haze formation and other forms of air pollution.

Climate change could alter the dispersion of primary pollutants, especially PM, and intensify the formation of secondary pollutants, such as ozone. In addition, meteorological variables like temperature, humidity, and wind characteristics influence the emission, transport, dispersion, chemical transformation, and deposition of environmental pollutants [103].

## 4. ncRNAs as Part of EVs Cargo

RNA is part of the EVs’ cargo and is at the center of many functions attributed to EVs since it can alter gene expression and function in recipient cells. EVs are a heterogeneous group of vesicles released from cells under physiological and pathological conditions [104,105] that contain a cargo of bioactive molecules reflecting their parental cells and can modulate recipient cells’ behavior [106,107]. EVs are now considered an additional mechanism for intercellular communication, enabling cells to exchange information through proteins, lipids, and genetic material [108]. EVs are released from cells into the extracellular space and can be found in all biological fluids: blood [109], urine [110], saliva [111], cerebrospinal fluid [112], breast milk [113], bronchoalveolar lavage fluid (BALF) [114] etc. Furthermore, EV’s biogenesis has been a significant evolutionary advancement because the cargo encapsulated inside the vesicular structures is protected from the degradation of ribonucleases, deoxyribonucleases, and proteases that are abundant in the extracellular space. These enzymes cannot traverse the EVs lipid bilayer [115]. The uptake of EVs by recipient cells can be either selective (via ligand-receptor interaction) or non-selective [116]. EVs are generally classified according to certain intrinsic properties, including density, size, and biogenesis processes. They are generally divided into three subtypes according to their biogenesis mechanism: exosomes, microvesicles and apoptotic bodies [117]. The International Society for Extracellular Vesicles (ISEV) currently encourages the use of the term “extracellular vesicles” as a generic term for all secreted vesicles, considering the lack of consensus for the identification of specific markers to distinguish between the different subtypes of EVs [118]. Based on their size, we can distinguish: ‘small EVs’ (sEVs) and ‘medium/large EVs’ (m/lEVs), which have defined ranges of <100 nm or <200 nm for small EVs and >200 nm for large and/or medium EVs, respectively [118].

EV-associated RNAs (EV-RNA) consist of RNA fragments of different sizes and include mRNA, pre-miRNA and mature miRNA precursors, snoRNA, rRNA, tRNA, lncRNA, piRNA, and mitochondrial RNA [119]. Initially, the focus on EVs cargo was on proteins, but the discovery 2007 of biologically active RNA particles in small EVs amplified the potential role of EVs in biology [120]. Currently, the Vesiclepedia database includes more than 27,000 entries for mRNAs and more than 10,000 entries for non-coding RNAs [121]. The role of non-coding encapsulated RNAs in EVs is emerging, especially in liquid biopsy diagnostics, a promising non-invasive alternative to traditional methods of diagnosis and prognosis [122]. The liquid biopsy method is based on identifying clinically relevant biomarkers. It allows for early diagnosis and monitoring of diseases from their earliest stages, which would bring enormous benefits to human health.

It has been shown that the ncRNA content inside EVs is higher than in the cells from which they originate, in contrast to the protein and lipid content that reflect the parent cells [123,124]. This has led researchers to hypothesize the existence of highly selective RNA-loading mechanisms inside EVs. The interactions with RNA-binding proteins (RBPs), such as argonaute 2 (AGO2), ALIX and annexin A2, appear to be crucial to explain the highly controlled and specific process of selective ncRNA packaging in EVs [125,126,127,128,129]. It must be noted that RBPs constitute about 25% of the protein content of EVs [130]. Furthermore, it was shown that post-transcriptional modifications at the 3′ end of miRNAs could also influence their selective loading into EVs [129]. RNA-EVs are divided into three types: RNAs with known functions, such as intact mRNA and miRNA; RNAs with probable but unproven intercellular mediator functions, such as piRNA; and, thirdly, RNA fragments (e.g., tRNA and mRNA and rRNA fragments) with unknown functions, which may be non-functional degradation products [119]. EVs could also play a potential role in drug therapy due to their cargo-protecting characteristic, as they could be used to deliver drugs in a targeted manner to cells (e.g., cancer cells). This opens the door to potential RNA-based therapies, including siRNAs, miRNAs, antisense oligonucleotides, mRNAs, guide RNAs and self-amplifying RNAs. The advantages of the therapeutic use of EVs include their high biocompatibility and stability and their limited immunogenicity [130].

## 5. The Effects of Pollutants on the Production, Release, and Cargo of Circulating EVs

Environmental pollutants contribute to the pathogenesis of numerous human diseases, with chronic inflammation and oxidative stress as common hallmarks. Emerging evidence suggests that EVs may play a role in the relationship between environmental pollutants and the pathogenesis of chronic systemic diseases [131]. Physiologically, EVs from the pulmonary region exhibit protective effects against stress signals since they participate in maintaining pulmonary homeostasis. However, air pollution alters the composition of pulmonary EVs, leading to a dysregulation of the EVs cargo and increasing their production and release [132]. One of the functions of the respiratory epithelium is to defend the body against pathogens or environmental pollutants. In addition, it is involved in the regulation of immune responses, as well as in tissue repair and remodeling after injury [133]. However, repeated exposure of the respiratory epithelium to air pollution induces chronic airway inflammation that can lead to the release of inflammatory molecular signals in the circulatory system [134]. For instance, exposure to PM has been shown to lead to an increased release of EVs, especially in overweight subjects [135].

The role of alveolar macrophages and the respiratory epithelium is to rapidly eliminate inhaled harmful substances and thus maintain an anti-inflammatory state. EVs from alveolar macrophages contribute to this anti-inflammatory state by providing a suppressor of cytokine signaling (SOCS) 1 and 3 to epithelial cells. Studies have been conducted on mice exposed to cigarette smoke extract (CSE) and human smokers. Both showed reduced SOCS concentrations in BALF compared to non-smoking controls, confirming a loss in smokers of the anti-inflammatory status derived from EVs [136]. Exposure to CSE, PM and other air pollutants has been shown to alter transcriptomic (miRNAs) composition and increase the release of circulating EVs derived from endothelial cells and immune cells, as well as pro-inflammatory molecules [137,138,139]. Bronchial epithelial cells and lung fibroblasts have been shown to release CD63 + CD81 + and TF + EVs in response to respiratory toxicants [140]. Thus, exposure to environmental pollutants appears to shift the functionality of EVs secreted by mononuclear, epithelial, and endothelial cells from an anti-inflammatory to a pro-inflammatory phenotype. Exposure to environmental agents may ultimately trigger epigenetic changes because abnormal ncRNA expression within EVs may induce developmental changes or lead to disease progression.

## 6. ncRNAs and Links to Respiratory Diseases

Pollution is known to cause and aggravate several chronic respiratory diseases. The World Health Organization has ranked air pollution as the most common environmental cause of premature death [141]. Several studies have suggested that ncRNAs inside EVs are involved in respiratory diseases caused by air pollution. For example, one study found that exposure to PM increased the expression of miR-222-3p in EVs isolated from bronchial epithelial cells [142]. This miRNA was shown to target genes involved in oxidative stress and inflammation, suggesting that it may contribute to the development of airway inflammation and damage caused by PM exposure. In another study, miR-146a and miR-146b were significantly elevated in bronchoalveolar lavage fluid (BALF) and lung tissue homogenate from mice exposed to PM 2.5 [143]. Furthermore, it was seen that in bronchial epithelial cells treated with PM 2.5, there was the secretion of EVs that increased bronchial smooth muscle cell contractility and contributed to airway hyperresponsiveness [144].

Other studies have also reported that EVs, carrying ncRNAs in their cargo, are functionally involved in cancer initiation, progression and the development of metastases [145]. Exposure to PM 2.5 induced differential expression of vesicular miRNAs, leading to tumour development [146]. ncRNAs as cargo of EVs can be used as biomarkers of lung cancer (LC) because it was discovered that certain miRNAs are dysregulated in LC patients, including miR-21, miR-23b-3p, miR-10b-5p, miR-139-5p, miR-200b-5p, miR-378a, miR-379 and miR-4257 [147,148].

lncRNAs regulate certain biochemical and cellular processes, such as gene expression, RNA splicing and ligand-receptor engagement, which in turn mediate the pathogenesis of respiratory disorders [149]. They have also emerged as novel master regulators of initiation, progression, and response to therapy in various cancers. lncRNA HOX transcribed antisense RNA (HOTAIR) represses gene expression, and its high expression in LC correlates with metastasis and poor prognosis [150]. MALAT1 is one of the most highly valued lncRNAs in lung cancer. It modulates miR-124/STAT3 to promote carcinogenesis [151] and the miR-204/SLUG axis to enhance epithelial-mesenchymal transition (EMT) and lymph node metastasis [152]. Another widely evaluated oncogenic lncRNA in lung cancer is XIST because its downregulation has been shown to inhibit tumor growth via the upregulation of E-cadherin and downregulation of Bcl-2 [153].

In COPD, there is severe airway inflammation with consequent damage to the lung parenchyma. This leads to the destruction of the alveolar wall and rarefaction of the alveolar sacs, resulting in breathing difficulties and reduced lung function (irreversible airway obstruction) [154]. Air pollution is a significant risk factor for COPD, and exposure to particulate matter (PM) has been shown to induce inflammation and oxidative stress in the lungs, which can contribute to the development and progression of COPD. Prevention is the key to minimizing risk factors, such as air pollution and cigarette smoke. It has been shown that miR-210 and miR-218 expression is significantly higher in EVs released from primary human bronchial epithelial cells (HBEC) after exposure to cigarette smoke. This promotes airway fibrosis in the pathogenesis of COPD [155]. It was subsequently observed that following exposure to cigarette smoke, EVs showed a reduced expression of specific miRNAs, including let-7d, miR-191, miR-126 and miR125a, promoting apoptotic cell clearance [156]. Most studies report a global downregulation of miRNAs in response to cigarette smoke, especially from human alveolar macrophages, probably by modification of DICER, an RNA endonuclease involved in miRNA maturation [157,158]. The expression of some miRNAs, such as miR-638 and miR-101, appear to correlate with the severity of regional emphysema [159,160]. Among lncRNAs, increased expression of SCAL1 was seen in airway epithelial cells as part of the oxidative stress response to cigarette smoke exposure [161].

Asthma is a chronic inflammatory disease characterized by airway remodeling and bronchial hyperresponsiveness caused by both genetic factors and environmental stress factors. Air pollution is a major environmental factor that can exacerbate asthma symptoms. Exposure to air pollutants, such as particulate matter, ozone, nitrogen dioxide, and sulfur dioxide, can trigger inflammation in the airways, leading to asthma attacks. EVs derived from bronchoalveolar lavage fluid of asthmatic patients contained higher levels of certain miRNAs than healthy controls, suggesting that these EVs may play a role in the development of asthma [162,163]. ncRNAs play a key role in the onset and progression of asthma by regulating gene transcription [164]. miRNAs influence asthma pathogenesis by regulating immune cells, bronchial epithelial cells and airway smooth muscle cells (ASMCs). It has been shown that miR-1, miR-18a, miR-21, miR-146a, miR-155, miR-210 and miR-1248 can regulate T-cell function and the production of Th2 cytokines involved in the pathogenesis of asthma [165,166,167]. A significantly reduced expression of let-7a, miR-21, miR-133a, miR-155, miR-328 and miR-1248 was found in exhaled breath condensates from asthmatic subjects compared to healthy subjects [163]. Among lncRNAs, it was shown that lncRNA-MEG3 could competitively adsorb miR-17 in CD4+ T cells of asthmatic patients [168]. lncRNA MALAT1, on the other hand, adsorbs miR-155 by negatively regulating it and altering the Th1/Th2 balance among CD4+ T cells [169]. Another lncRNA under investigation is lncRNA PVT1, which, if over-regulated, could inhibit miR-149 expression in bronchial epithelial cells, promoting airway inflammation and cell barrier destruction, thus accelerating the development of asthma [170] (Table 2).

## 7. ncRNAs as Mediators of the Crosstalk between Air Pollution and Cardiovascular Diseases

Cardiovascular diseases (CVDs) are a group of disorders affecting the heart and the blood vessels, such as heart failure, coronary heart disease and acute myocardial infarction (AMI) and represent one of the leading causes of morbidity and mortality in the world [171].

Besides genetic causes, increasing compelling evidence has demonstrated that exposure to environmental air pollution, especially particulate matter (PM), significantly contributes to the development of CVDs and acute cardiac events like stroke, likely by triggering pulmonary inflammation, systemic inflammation, autonomic nervous system imbalance, oxidative stress, endothelial dysfunction and prothrombotic changes [172,173,174,175,176]. Accordingly, it has been shown that short-term exposures to PM and nitrogen oxides are consistently associated with increased risks of hypertension and triggering of myocardial infarction (MI) and stroke. In contrast, long-term exposures are largely associated with increased risk of atherosclerosis, incident MI, hypertension, and incident stroke and stroke mortality [177].

In recent years, researchers have started to investigate the underlying epigenetic component of CVDs. NcRNA-mediated gene regulation has been proposed to play a key role in the possible correlation between air pollutant exposure and the risk of CVDs [178,179,180]. It has been well established that short and long ncRNAs are involved in cardiac development and physiological functions. Moreover, they significantly contribute to the development and progression of various CVDs [181,182,183].

A cohort study by Bollati et al. analyzed male workers’ miRNA profile in peripheral blood leukocytes after a three-day exposure to ambient PM rich in metals. The examined subjects were not affected by CVDs, or other conditions in which systemic inflammation occurs, such as cancer and respiratory diseases. A qRT-PCR analysis revealed a significant increase in miR-21 and miR-222. Moreover, a positive correlation was found between the levels of miR-21 and 8-hydroxy-guanine (8-OH-dG), suggesting a relationship with oxidative stress [184]. Similarly, mir-222 has been found in higher concentrations in the extracellular fraction of saliva in schoolchildren following recent exposure to ultrafine particles (UFP) [185]. Both miR-21 and miR-222 are highly expressed in the cardiovascular system, regulating critical biological pathways. For instance, miR-21 has been reported to exert a cardioprotective role [186].

On the contrary, they are dysregulated in numerous CVDs, suggesting they could be used as promising cardiovascular biomarkers and novel therapeutic targets/agents in CVDs, including air pollution-correlated ones [187,188]. However, in a cohort of 153 elderly males, an inverse relationship was found between eight leukocyte miRNAs, including miR-21 and miR-222, and PM 2.5 exposure. According to the authors, considering the activity of the predicted target mRNAs, this negative association should result in increased inflammation, endothelial dysfunction, and atherosclerosis [189]. The contradictory results could be due to differences in the methodologies used by the two research teams, the particle composition, the time of exposure and/or the characteristics of the participants.

Chen and colleagues observed that in healthy young subjects, short-term exposure to PM was positively associated with the expression (mRNAs, proteins, or both) of cytokines involved in inflammation, coagulation, and endothelial dysfunction, including IL1, IL6, TNF, toll-like receptor 2, coagulation factor 3, and endothelin 1. On the contrary, it was negatively associated with the expression of miRNAs predicted to target these cytokines’ mRNAs, such as miR-21-5p, miR-187-3p, miR-146a-5p, miR-1-3p, and miR-199a-5p. Thus, the authors suggested that a possible correlation between PM exposure and CVD development might be mediated by miRNAs controlling specific cytokine expression [190].

Another interesting line of research regarding the correlation between exposure to environmental contaminants and increased CVD risk focused on investigating EVs’ miRNA content. Numerous studies have shown that exposure to ambient air pollutants, including fine particulate matter (PM 2.5), coarse particulate matter (PM 10), ozone (O3), and nitrogen oxides (NOx), was significantly associated with changes in the expression of some circulating miRNAs, including miR-26a-5p, miR-146a-5p, miR-150–5p and miR-21-5p, some of which may mediate their effects on the downstream inflammation, coagulation, and blood lipid biomarkers [134,139,190,191,192,193,194,195,196].

In this context, Bollati and coworkers investigated whether PM and metal-rich PM may alter microvesicles (MVs) content, finding that miR-128 and miR-302 were significantly overexpressed after three days of PM exposure, compared with the beginning of the working week [134]. Both miRNAs regulate gene expression linked with CVDs, including coronary artery disease, cardiac hypertrophy, and heart failure pathways, suggesting MV-associated miRNAs as mediators of air pollution toxicity [134]. Furthermore, the effects of metal-rich PM have been evaluated in a subset of the same study population, identifying four PM-sensitive miRNAs (miR-29a-3p, miR-146a-5p, miR-421, and let-7 g-5p) that were differentially expressed compared to control samples and seem to favor a pro-inflammatory response, potentially contributing to several diseases, including CVDs and respiratory system disorders [139,191]. Similarly, Rodosthenous and colleagues investigated the relationship between short-, intermediate-, and long-term exposures to PM and the EVs-miRNome in a cohort of healthy adults. They observed long-term exposure to PM 2.5 increased some EVs’ miRNAs in serum. The predicted target genes, including interleukin 6 (IL-6), C-X-C motif chemokine ligand 12 (CXCL12), vascular cell adhesion molecule 1 (VCAM-1), a cluster of differentiation 40 (CD40), and platelet-derived growth factor subunit beta (PDGFB), are linked to CVD-related signaling pathways, such as oxidative stress, inflammation, atherosclerosis, and cardiac hypertrophy [193]. These results were confirmed by a subsequent study by the same authors, where it was demonstrated that an increase of multiple EVs’ mRNAs in serum from elderly males could modify the association between PM 2.5 and systolic blood pressure by targeting proteins involved in essential cardiovascular functions [194]. These results aligned with previous data from Motta et al., which demonstrated that miRNAs are responsible for the association between exposure to particulate air pollution and increased blood pressure, a well-established risk factor for cardiovascular disease [197].

Pergoli and colleagues observed that short-term exposure to PM 10 was associated with an increased release of EVs, especially from monocytes/macrophages (CD14+) and platelets (CD61+), as well as high fibrinogen levels in overweight/obese people [192]. The analysis of these EVs’ content revealed that nine miRNAs were downregulated in response to PM exposure. As highlighted by integrated network analysis, five of them, i.e., let-7c, miR-106a, miR-185, miR-331, and miR-652, modulate cardiovascular-linked processes, including fibrinogen levels, suggesting an association between extracellular miRNA released after short-term exposure to PM and increased coagulation [192]. Therefore, air pollutant exposure may affect the EVs’ content and downstream effects on target cells. Most recently, a change of small EV (sEVs) subpopulations released from the respiratory system has also been observed in response to PM 2.5 exposure, increasing CD63/CD81/CD9-positive particles [196]. In particular, the authors observed that these sEVs contained miR-421, which contributed to cardiac dysfunction by targeting the cardiac angiotensin-converting enzyme 2 (ACE2), suggesting the sEVs’ miRNome as responsible for the crosstalk between lung and heart upon PM 2.5 exposure [196] (Table 2).

## 8. ncRNAs and Neurodegenerative Diseases

Neurodegenerative diseases (NDDs), including Parkinson’s disease (PD), Alzheimer’s disease (AD), and other dementia disorders, affect millions of people worldwide and are caused by the progressive degeneration and death of select populations of neurons. This process leads to debilitating conditions such as cognitive, motor, and behavioral impairments [198]. Recent studies indicate that air pollution may also adversely affect the brain, contributing to the etiopathogenesis of neurodegenerative diseases. There is growing evidence that traffic-related air pollution (TRAP) exposure might lead to the development and progression of neurodegenerative disorders [199]. A study conducted in the U.S. reported that the risk of developing dementia for residents in areas with high PM 2.5 was increased by 92% compared to those who lived in areas with lower levels of particulate matter [200]. In addition, another study conducted on mice demonstrated the induced glutaminase-containing EVs release from microglial cells. The increment of glutamate is associated with neurodegeneration [201]. Several studies suggest that exposure to air pollution might be associated with PD, a long-term degenerative disorder of the central nervous system that mainly affects the motor system. Different expression levels of several miRNAs can be used as potential diagnostic biomarkers for PD. Particularly, EV-derived miRNAs are increasingly used to diagnose the disease [202]. A study on blood-derived EVs in PD reported higher miR-34a-5p levels in PD patients compared to controls, whereas other similar studies on serum EV-derived miRNAs found upregulated miRNAs, such as miR-22, miR-23a, miR-24, andmiR-222, and a downregulated miRNA, miR-505, in PD patients [203,204]. Also, EVs isolated from the cerebrospinal fluid (CSF) are a promising source for PD diagnostic markers. The qRT-PCR analysis showed differences in the miR-181a-5p, miR-181b-5p, miR-9-5p and let-7b in PD patients versus control comparisons [203].

Various studies have demonstrated a strong association between air pollution and AD. In this neurodegenerative disease, the amyloid-beta protein (Aβ) dysregulation leads to aggregates of amyloid fibrils and neurotoxicity. A German study showed that exposure to PM 2.5 and NOx was associated with an increased risk for AD [205]. Moreover, a study conducted in London concluded that residents with the most exposure to nitrogen dioxide and PM 2.5 were the most likely to be diagnosed with dementia, with associations more consistent for AD [206]. Compared to healthy controls, a study on plasma-derived EVs of AD patients reported downregulation of miR-342-3p, miR-451 and miR-21-5p. On the contrary, other studies revealed several EV-derived miRNAs, such as miR-29a, -451a, -125b, -582-5p, -106a-5p, and -106b-5p, were upregulated in AD patients, compared to healthy controls [207,208,209].

**Table 2:** ncRNAs role in environmental stress response and their involvement in respiratory, cardiovascular, and nervous system pathogenesis. The table also summarizes their possible contribution as biomarkers.

Disease	Type of Disease	Environmental Stressors	ncRNA	Description	Refs.
Respiratory diseases		Exposure to PM	Increased expression of miR-222-3p in EVs isolated from bronchial epithelial cells	miRNA involved in oxidative stress and inflammation; contribution to the development of airway inflammation	[142]
		Exposure to PM 2.5	miR-146a and miR-146b	Significantly elevated BALF and lung tissue homogenate from mice	[143]
		Bronchial epithelial cells treated with PM 2.5	EVs carry ncRNAs in their cargo	Secretion of EVs that increased bronchial smooth muscle cell contractility and contributed to airway hyperresponsiveness	[144]
	Lung cancer (LC)	Exposure to PM 2.5	miR-21, miR-23b-3p, miR-10b-5p, miR-139-5p, miR-200b-5p, miR-378a, miR-379 and miR-4257	Dysregulated ncRNAs as EVs cargo can be used as biomarkers of LC	[147,148]
	Lung cancer (LC)		lncRNA HOTAIR	HOTAIR high expression in LC correlates with metastasis and poor prognosis	[150]
	COPD	Exposure to cigarette smoke	miR-210 and miR-218	Their expression is highest in EVs released from HBECs. This promotes airway fibrosis in the pathogenesis of COPD	[155]
		Exposure to cigarette smoke	let-7d, miR-191, miR-126 and miR125a	EVs showed a reduced expression of these miRNAs, promoting apoptotic cell clearance	[156]
		Exposure to cigarette smoke	lncRNA SCAL1	Increased expression of SCAL1 in airway epithelial cells as part of the oxidative stress response	[161]
	Asthma	Air pollution can exacerbate asthma symptoms	miR-1, miR-18a, miR-21, miR-146a, miR-155, miR-210 and miR-1248	They can regulate T-cell function and the production of Th2 cytokines involved in the pathogenesis of asthma	[164,165,166]
	Asthma	Air pollution can exacerbate asthma symptoms	let-7a, miR-21, miR-133a, miR-155, miR-328 and miR-1248	Reduced expression in exhaled breath condensates from asthmatic subjects	[167]
	Asthma	Air pollution can exacerbate asthma symptoms	lncRNA-MEG3 MALAT1 PVT1	Altered expression of these lncRNAs compared to controls	[168,169,170]
Cardiovascular diseases		Three-day exposure to ambient PM rich in metals	miRNA profile of peripheral blood leukocytes	Increased levels of miR-21 and miR-222 and positive correlation with 8-OH-dG level and oxidative stress	[182]
		Exposure to ultrafine particles	miRNAs from the extracellular fraction of saliva in schoolchildren	Increased concentration of miR-222 and possible positive correlation with CVDs	[185]
		Exposure to PM 2.5	Eight leukocyte miRNAs	Reduced levels of miRNAs, including miR-21 and miR-222. Increase in inflammation, endothelial dysfunction, and atherosclerosis processes	[189]
		Short-term exposure to PM	miR-21-5p, miR-187-3p, miR-146a-5p, miR-1-3p, and miR-199a-5p	Reduced levels of miRNAs and increase of the targeted cytokines, which are involved in inflammation, coagulation, and endothelial dysfunction	[189]
	Coronary artery disease, cardiac hypertrophy, and heart failure	Three-day exposure to PM	miR-128 and miR-302 from circulating microvesicles	miRNAs overexpression and possible positive correlation with CVD development	[190]
		Exposure to metal-rich PM	miR-29a-3p, miR-146a-5p, miR-421, and let-7 g-5p	Altered expression of miRNAs and positive correlation with a pro-inflammatory response contributing to CVD development	[191]
		Short-term exposure to PM 10	miRNAs from monocytes/macrophages (CD14+) and platelets (CD61+)-derived EVs	Downregulation of miRNAs expression and increase of coagulation	[192]
		Long-term exposure to PM 2.5	miRNAs from serum EVs	Increase of EVs’ miRNAs, whose targets are involved in some CVD-related signaling pathways, such as oxidative stress, inflammation, atherosclerosis, and cardiac hypertrophy	[193]
		Long-term exposure to PM 2.5	miRNAs from serum EVs	Increase of EVs’ miRNAs, whose targets are involved in essential cardiovascular functions	[194]
		Exposure to PM 2.5	miR-421 from small EVs released from the respiratory system	Positive correlation with cardiac dysfunction	[196]
Neurodegenerative diseases	PD	Exposure to air pollution	miR-34a-5p, miR-181a-5p, miR-181b-5p, miR-9-5p and let-7b	Altered expression of these miRNAs compared to controls	[203]
	PD	Exposure to air pollution	miR-22, miR-23a, miR-24, miR-222, mir-505	Altered expression of these EVs’ miRNAs	[204]
	AD	Exposure to air pollution	miR-342-3p, miR-451 and miR-21-5p	Downregulation of EVs’ miRNAs expression	[207]
	AD	Exposure to air pollution	miR-29a, -451a, -125b, -582-5p, -106a-5p, and -106b-5p	Increased expression of EVs’ miRNAs	[208,209]
	VD	Exposure to air pollution	miR-135a, miR-193b, -23a, -29a, -130b	Altered expression of these EVs’ miRNAs	[207]
	FTD	Exposure to air pollution	miR-204-5p and mirR-632	Downregulation of miRNAs expression	[207]

Furthermore, exposure to air pollution is also associated with other types of dementia disorders, including vascular dementia (VD) and frontotemporal dementia (FTD) [210,211]. VD is dementia caused by reduced blood flow in various brain regions. In contrast, FTD is a rare type of dementia characterized by progressive nerve cell loss in the frontal and temporal lobes of the brain. Recent studies have shown that EV-derived miRNAs are involved in the pathogenesis of dementia disorders, making them valuable biomarkers for diagnosing dementia. Serum levels of EV-derived miR-135a increased, whereas levels of EV-derived miR-193b, -23a, -29a, -130b decreased in VD patients versus healthy controls. EVs isolated from FTD patients’ cerebrospinal fluid (CSF) showed downregulated expression of miR-204-5p and mirR-632 compared to healthy controls [207] (Table 2).

## 9. Conclusions and Future Perspectives

Poor air quality has become a significant threat to public health globally, and it is estimated to cause approximately 7 million deaths each year, as it increases the risk of a wide range of diseases. Considering its health impacts, conducting biological impact assessments of air pollution has thus become an urgent task for public health practitioners [212].

Growing evidence highlights the clear connection between environmental pollutants and human pathologies. Nevertheless, the mechanisms linking air pollutant exposure with mediating biological effects in cells and disease onset have not yet been fully elucidated.

**Figure 1:**
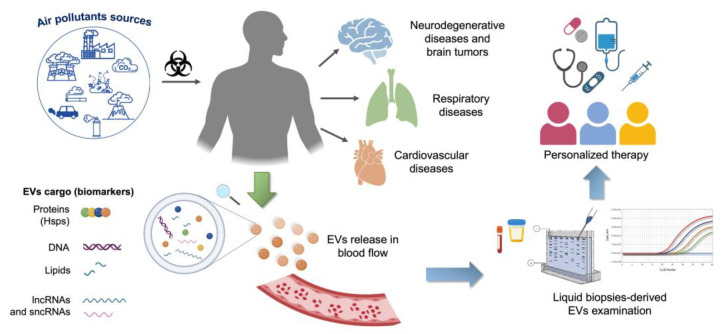
Schematic representation of the benefits of ncRNA in liquid biopsy in the study of diseases associated with air pollutant exposure. ncRNAs can be found in blood-derived EVs and represent reliable prognostic and predictive markers, useful in screening, diagnosis, and EV-based personalized therapy.

In response to environmental stresses, cells produce multiple mediators secreted into the extracellular environment via EVs [213,214,215]. Among these mediators, ncRNAs can determine multiorgan and systemic effects due to their ability to affect numerous genetic targets and manipulate the gene expression of target cells. Moreover, circulating EVs can be detected in the plasma of subjects exposed to particulate air pollutants, to identify a possible predictive role of peripheral blood ncRNA signatures in human diseases. Evaluating different EV-carried ncRNA signatures could represent a good opportunity to find new biomarkers for the diagnostics and subsequent stratification, prognostics and evaluating treatment efficacy in patients with air pollutant exposure-associated diseases. In addition, ncRNAs could be used to optimize EV-based therapeutic approaches, such as personalized medicine (Figure 1).

## Figures and Tables

**Table 1:** Classification of ncRNAs.

Types		Size	Function
Housekeeping RNAs	rRNAs	120–5000 nts	Proteins synthesis
	tRNAs	70–100 nts	Proteins synthesis
	snRNAs	~150 nts	Splicing of mRNAs
	snoRNAs	60–300 nts	Modification of rRNAs
	TERC	400–600 nts	Template for telomere replication
Regulatory RNAs	siRNAs	20–25 nts	Regulation of gene expression at the post-transcriptional level
	miRNAs	21–23 nts	Regulation of gene expression at the post-transcriptional level
	piRNAs	26–31 nts	Regulation of gene expression at epigenetic, transcriptional, and post-transcriptional level
	lncRNAs	>200 nts	Regulation of gene expression at epigenetic, transcriptional, and transcriptional level

## Data Availability

No new data were created.
